# Structural, optical, and cytotoxicity studies of laser irradiated ZnO doped borate bioactive glasses

**DOI:** 10.1038/s41598-023-34458-4

**Published:** 2023-05-05

**Authors:** Ahmed R. Ghazy, B. M. Elmowafy, A. M. Abdelghany, T. M. Meaz, R. Ghazy, R. M. Ramadan

**Affiliations:** 1 Physics Department, Faculty of Science, Tanta University, Tanta, 31527 Egypt; 2 Spectroscopy Department, Physics Research Institute, National Research Centre, 33 Elbehouth St., Dokki, Giza, 12311 Egypt; 3 Microwave Physics and Dielectrics, Physics Research Institute, National Research Centre, 33 Elbehouth St., Dokki, Giza, 12311 Egypt

**Keywords:** Biophysics, Biotechnology

## Abstract

Borate glasses (BG) doped with different amounts of ZnO (0–0.6 mol%) were formed by the traditional melt quenching technique. The different glasses so made were characterized using different characterization techniques such as X-ray diffraction (XRD), Fourier transform infrared spectroscopy (FTIR), scanning electron microscope (SEM), and UV–Vis absorption optical properties. The XRD patterns showed an amorphous structure with one broad peak at 2*θ* = 29°, while the phonons bands were studied in terms of the FTIR bands. Optical properties of the glasses were studied using UV–Vis absorption spectra in the range 190–1100 nm, in which the prominent band lies at about 261.5 nm of peak position, from which the bandgab (E_g_) was calculated from its edge using Tauc’s plot, with E_g_ ~ 3.5 eV. The laser irradiation showed no significant changes in the absorption bands, despite a significant change observed in the amorphous behavior in the XRD pattern. The cell viability was performed for two samples of the BG and 0.6 mol% ZnO doped using 3-(4,5-dimethyl-2-thiazolyl)-2,5-diphenyl-2H-tetrazolium bromide (MTT) assay method. The result showed better cell viability and low toxicity. So, ZnO doped BG can be used in various biomedical applications.

## Introduction

In the last decades, the field of biomaterials has grown at an incredible rate, leading to the development of bioactive materials, which may elicit specific and predictable responses from cells and tissues^1^. The discovery of Hench glass in 1969 constituted for the first time a revolution in the history of biomaterials^2,3^. The mechanical, bioactive, and structural properties of the bioactive glasses are largely based on synthesis procedures, composition, particle size, crystallization, etc. Bioactive glasses are made using several approaches. The most common method for making bioactive glasses is the traditional melt-quenching method, in which all the components are well mixed in a ball mill before being melted at an elevated temperature^4^. In the melt-quenching process, a volatile part such as B_2_O_3_ gets evaporated out^5–7^_._

In constructing a bioactive glass, understanding how the physicochemical structures of these materials influence their characteristics is critical, as it allows the material to be adapted for particular applications. Each component influences the bioactive glass's performance. Calcium, for example, promotes osteoblast development and apatite layer precipitation^8,9^. Na_2_O. K_2_O. MgO. CaO. P_2_O_5_-based glasses found to constitute a promising material for bioactive applications such as bone repair, tissue regeneration in the human body, etc.^10–13^. Borate glasses quickly release significant amounts of boron, leading to a high concentration of local boron near the glass. As a result, compared to silicate glasses, the degradation and sintering behavior of borate/borosilicate glass is more controllable^14–16^. In borate glasses, the boron oxide appears in BO_3_ and BO_4_ in a network structure that forms 'super structural' units (pentaborate, boroxol ring, diborate, or tetraborate groups), depending on the composition and the kind of added glass modifiers^17–20^.

The glass exhibits nonlinear change in its physical properties when one alkali ion is replaced with another alkali content at a constant amount, resulting in a mixed alkali effect (MAE)^21^. Incorporation of zinc oxide in glass structures is expected to acts as a intermediate oxide either as network former or as network modifier^22,23^. Glass composition is modified by introducing the ‘dopants’ to the glass network to form the desired glass where it can be bioactive, bioresorbable, and/or biodegradable. Dopants like Cu, Zn, In, Ba, La, Y, Fe, Cr, and Sr as ions lead to trigger the properties. ZnO/MgO additives have been shown to stimulate osteoblast proliferation, differentiation, and bone mineralization^24–27^. Zn is an essential trace element that is used by various metalloenzymes for structure, catalysis, or regulatory functions. Zinc is involved in bone metabolism, enhancing osteoblastic bone formation and preventing osteoclastic bone resorption, raising bone mass^28^. Nutritional zinc supplementation has been demonstrated to have preventive and therapeutic effects on bone loss induced by bone disorders^29,30^. Investigations have also demonstrated that small quantities of Zn induced early cell proliferation and enhanced differentiation of in vitro biocompatibility studies^31–33^. Methodologies like MTT and MTS are used to assess the viability and cytotoxicity of the glass samples because these methods generally measure cytotoxicity for bulk constructions indirectly by extracting the materials.

According to Saranti et al., boron oxide has a catalytic action that promotes bioactivity^34^. Neáková et al. developed mesoporous bioactive glass nanoparticles (MBGNs) based on the SiO_2_–CaO system using a micro-emulsion supported sol–gel method. Zn^2+^ ions were doped into MBGNs with 8 Mol% ZnO concentration (Zn-MBGNs). The findings investigated that the addition of zinc precursors had no effect on particle morphology, but enhanced their specific surface area when compared to MBGNs^35^. Lee et al. reported that bone implant and osteointegration utilizing a B_2_O_3_-based glass technology represents no toxicity. Bone implant and osteointegration using B_2_O_3_ based glass system are reported by Lee et al. without any toxicity^36^. Kolavekar et al. studied the optical properties of Pr_2_O_3_-doped multi-component borate glasses^37^, TeO_2_-doped lead borate glasses^38^, Li^+^ Ions doped zinc borate glass^39^ and Er^3+^ and Er^3+^/Yb^3+^ co-doped heavy metal borate glasses^40^ and showed the effect of the dopants on the photophysical properties of the borate glass.

Bioactive glasses are effective biomaterials for promoting angiogenesis in both hard and soft tissue engineering applications. Metallic ions like Cu^2+^, Ag^2+^, Mg^2+^, Zn^2+^, Fe^3+^, Sr^2+^, and Co^2+^ have been utilized as dopants in oxide glasses^41–43^. The presence of these ions in the glass network causes antibacterial agents, osteogenesis motivation factors, and angiogenesis enhancers^44,45^.

The effect of laser irradiation was studied for borosilicate glasses attracted much of scientisits attention because it can stimulate various microspheres that can be controlled within transparent materials due to non-linear optical absorption^46,47^. The novelty in the work is to study the effect of laser irradiation on the structure and optical properties of ZnO doped borate bioactive glass.

This study is aimed to investigate the effect of ZnO on the structure of bioactive borate glass using X-ray diffraction (XRD) and Fourier transform infrared spectroscopy (FTIR). The effects of laser irradiation on the selected glasses are studied in terms of their UV–Vis absorption spectra and XRD pattern. The cytotoxicity of ZnO-doped bioactive borate glass on cell viability is assessed in a contest to bioapplications.

## Experimental work

### Materials (glass preparation)

The glasses of compositions 6Na_2_O + 12K_2_O. + 5MgO + 20CaO + 4P_2_O_5_ + (53-x) B_2_O_3_ + xZnO (0 ≤ x ≤ 0.6 mol%), were prepared via traditional melt quenching method. Highly pure chemicals of orthoboric acid (a source for B_2_O_3_) and ammonium dihydrogen phosphate (a source for P_2_O_5_) supplied by Sigma Aldrich Co were used. Na_2_O, K_2_O, MgO, and CaO were added as carbonates provided by El Nasr Pharmaceuticals. All the chemicals were used as received. The chosen glass batches were mixed and melted in an electrical furnace at 1100–1150 °C and swirled to assure homogeneity, and then the melts were quenched and pressed between two steel plates at room temperature to obtain the glasses. The so-obtained glasses were characterized using XRD patterns, FTIR, and UV–visible absorption spectra. Images of the formed glasses are listed in the Table 1.

**Table 1:** Photo images of borate glass doped with different amounts of ZnO.

Sample	Base	0.1 mol% ZnO	0.2 mol% ZnO	0.3 mol% ZnO	0.4 mol% ZnO	0.6 mol% ZnO
Image						

### Characterization and analysis techniques

The various methods of analysis and typical settings that describe ZnO doped borate glass are recorded by utilizing the following instruments: (a) the X-ray diffraction pattern (XRD) was recorded in the range of 4° ≤ 2θ ≤ 70° using a Rigaku X-ray diffractometer ultima IV with CuKα radiation of wavelength λ = 0.154600 nm and steps of 0.02°. (b) Microstructure of the samples was examined using a scanning electron microscope (JEOL JSM-6510LV, USA), which used a focused electron beam (operating at 20 kV accelerating voltage) with a magnification up to 40,000 X, where the samples were coated with gold so the surface becomes conducting to measure the images. (c) A FTIR spectrometer (type Nicolet i10, Thermo Fisher Co.) was used to record FTIR spectra of the glasses over a 4000–400 cm^−1^ range, with a 2 cm^−1^ step resolution. Measurements were performed on powders dispersed in KBr in a 1:100 ratio in the form of thin pellets. (d) UV/Visible absorption spectra were recorded (in a range of 190 to 1100 nm) for the polished `samples, using a double beam (JASCO V570 UV/Vis./NIR) spectrophotometer, with air as a reference sample. All the glass samples were irradiated for 30 min by a laser beam (λ = 375 nm) with a 150 mV power.

The technique of 3-(4,5-dimethyl-2-thiazolyl)-2,5-diphenyl-2H-tetrazolium bromide (MTT) assay technique was used to assess cytotoxicity and proliferation was purchased from (Serva, Germany). Cells were seeded in well plates and cultured for 48 h to determine the IC50 for the different glasses and compared with the control sample (normal cell without glass). The glass was solubilized in dimethyl sulfoxide (DMSO) stoke before treatment. The reduction in cell growth was measured at (570 nm) (BioTek, Elx800, US) and the results were calculated as a percentage of control. Prism software was used to calculate the IC50 of glass concentrations as well as cell viability.

## Results and discussion

### XRD patterns and microstructure

**Figure 1:**
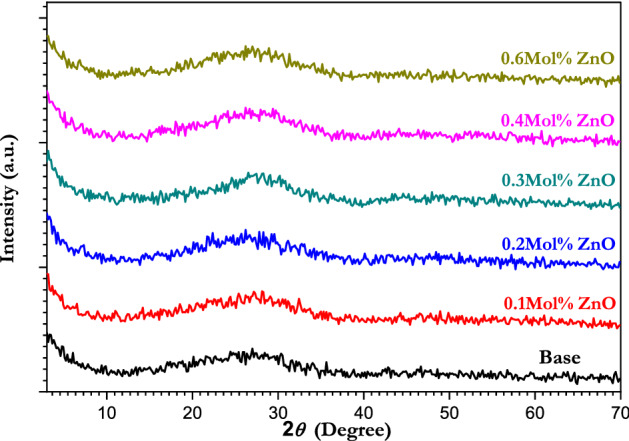
XRD patterns of different concentrations of ZnO doped borate glass in the range of 4° ≤ 2θ ≤ 70°.

Figure 1 presents XRD patterns of the ZnO doped borate glass, which reveal an amorphous structure for all the samples with a broad diffraction peak at 2*θ* = 29° of diffraction angle ‘2*θ’,* with no sharp peak in absence of any crystallites. The absence of clearly defined diffraction peaks confirms the samples' glassy nature and rules out the possibility of long-range atomic organization^48,49^. The absence of Bragg's peak and the amorphous glassy nature of all the glass samples were confirmed by X-ray diffraction patterns due to the presence of potassium and magnesium, in particular, which enhanced glass processability, making it easier to make glass without crystallization and use it in coatings, fibers, scaffolding and other applications^50^.

**Figure 2:**
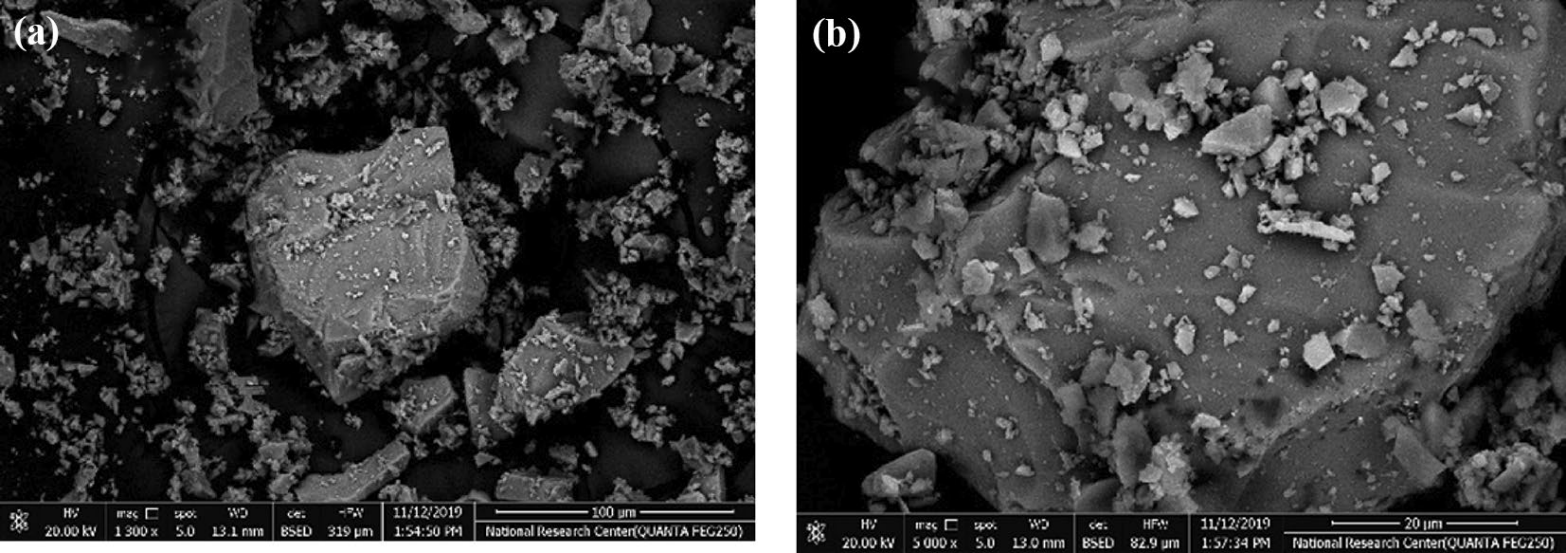
SEM images of borate glass doped with 0.6 mol% ZnO; (**a**) lower (**b**) higher magnifications.

Figure 2a, b represent SEM of borate glass doped with 0.6 mol% ZnO of lower and higher magnification which showed dispersed particles or grain-like structure appeared in the morphology of 0.6Mol% ZnO doped borate glass. SEM images indicate the domination of the amorphous structure of the borate matrix as a continuous phase. A good agreement with the suggested amorphous structure from the XRD pattern was demonstrated from the SEM images.

**Figure 3:**
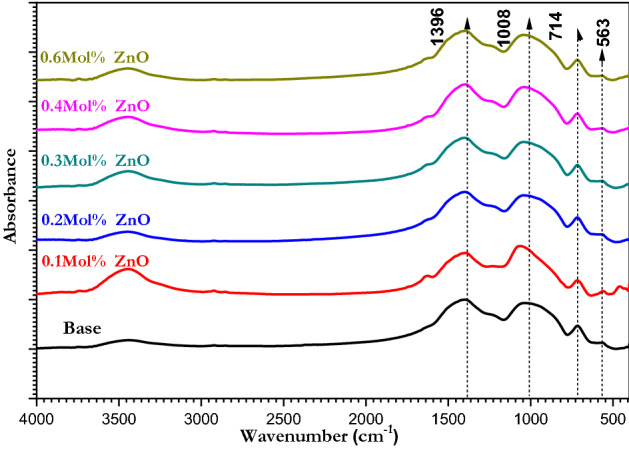
FTIR spectra of borate glasses doped with different concentrations of ZnO.

Figure 3 shows FTIR spectra of the ZnO doped borate glass where there were broad absorption bands that indicate the groups inside the network system. The interpretation of IR of borate glass is summarized as follows: The first broad band around 1396 cm^−1^ indicated for B–O asymmetric stretching vibration band of trigonal BO_3_. The second band at 1008 cm^−1^ pointed to the B–O bond stretching of the tetrahedral BO_4_. The third band positioned nearly at 714 cm^−1^ refers to the bending of B–O–B in trigonal BO_3_ units and the last band at 563 cm^−1^ points to the vibration of metal cations like ZnO. The bands face no changes when ZnO nanoparticles are added to the composition. The strong appearance of vibrational broad bands of triangular and tetrahedral for borate glass owing to the presence of the two alkali metals Na_2_O and K_2_O. Also, many little changes were investigated after the minor addition of ZnO nanoparticles which indicated the effect of the concentration of ZnO nanoparticles on the transformation process. The band appears in the far infrared region 425 cm^−1^ due to the stretching vibration of transition metal ions such as (Zn^2+^, and Mn^2+^). The other main vibrational modes mentioned before are due to the borate glass matrix^51^.

**Figure 4:**
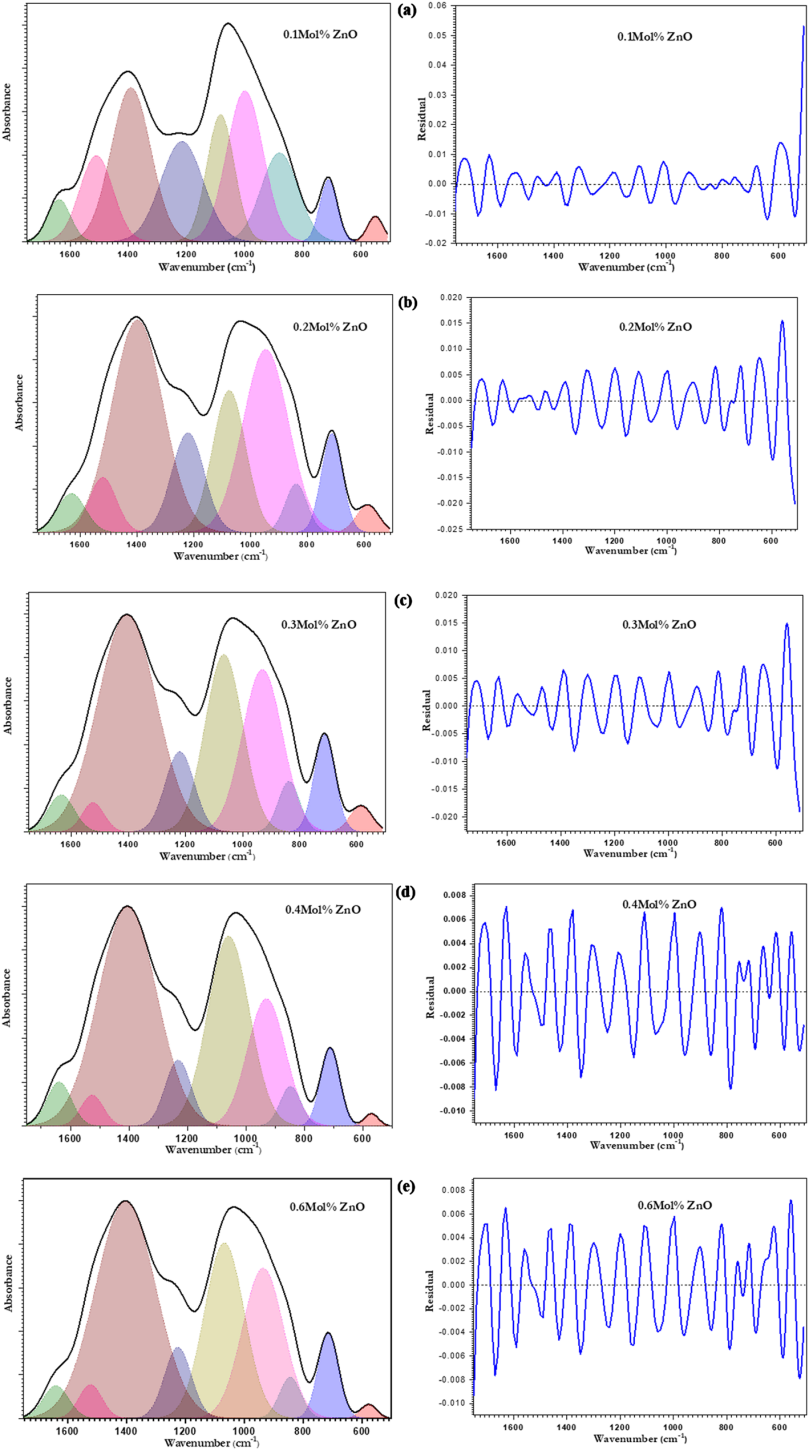
The deconvolution of FTIR spectra with its residual of ZnO doped borate glasses.

PeakFit4.12 computer program was used for the mathematical deconvoluted analysis technique (DAT) that was used to examine and analyze the collected FTIR spectral data of ZnO-doped borate glasses to obtain quantitative information about the internal changes inside the glass matrix. In the range of 1750–500 cm^−1^, the smeared overlapping bands of triangular and tetrahedral borate groups were resolved, while the region of 3200–3600 cm^−1^ reveals the vibrational modes of the OH group. The deconvoluted analysis was established using a small number of bands to resolve the spectra and then weaker bands were inserted to improve the fit. Figure 4a–e represents the deconvoluted analysis of ZnO-doped borate glasses and their residuals. The deconvolution process is based on the previous knowledge of the wavenumber of suggested vibrational groups and the second derivative of the spectrum that identifies the accurate position of peak maxima as previously described by different authors^52–56^. In such cases, the differences between the deconvoluted and measured spectrum can be minimized and plotted as shown in the residual curves.

**Figure 5:**
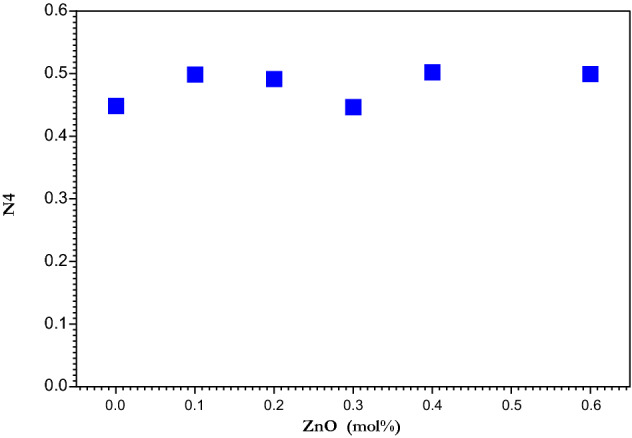
Variation of the fraction of four coordinated boron as a function of ZnO nanoparticles concentration.

Obtained N4 data of coordinated boron (N4 = BO_4_/(BO_4_ + BO_3_)) for ZnO doped borate glasses is represented in Fig. 5, which indicates boron atoms transformations occur inside the glass matrix after ZnO nanoparticles addition. It can be figured that the value of N4 increased by increasing the concentration of ZnO nanoparticles to 0.2 mol% and then faces two stages of decreasing and increasing. The creation of non-bridging oxygen NBOs can be correlated with the decrease in the N4 ratio with increasing in ZnO nanoparticles content in addition to other parameters. The negative charge on NBOs makes it easier for electrons to be excited at higher wavelengths^57^. Both ZnO and CaO act to reduce the BO_4_/(BO_4_:BO_3_) ratio and enhanced the glass network by increasing the number of non-bridging oxygen atoms^58^. In the glass structure, ZnO acts as a intermediate oxide either as network former or as network modifier^59^, where ZnO nanoparticles is a promising candidate as a modifier with a large band gap, allowing it to be used as a potential optical material^48,60^. As there was an unnoticeable change ZnO nanoparticles act as a modifier not as a former.

### Electronic absorption spectra

**Figure 6:**
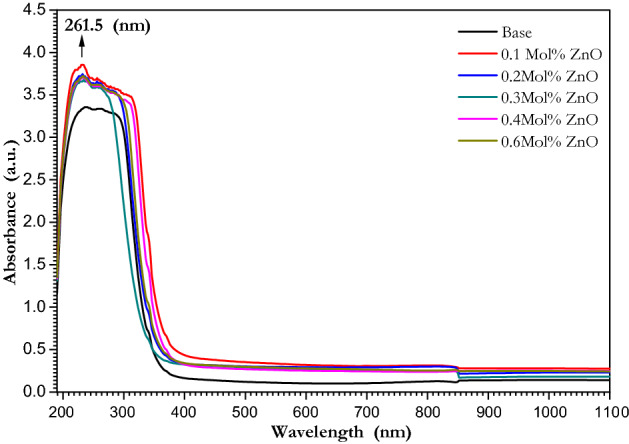
Absorption Spectra of ZnO doped borate glasses in the range of 190–1100 nm.

Ultraviolet–visible absorption spectroscopy represents the optical properties that give information about the electronic structure of the material^61,62^. UV–Vis. absorption spectra of ZnO doped borate glasses were explained in Fig. 6 in the wavelength range of 190–1100 nm. It can be figured that the absorption of UV–Vis arises from the higher wavelength to the lower wavelength of the glass materials. A high absorption band at the wavelength of 261.75 nm in the UV region of the spectrum was figured out for all doped glasses, which results from the unavoidable trace iron impurities in the raw materials during the glass formation^63^. While the broad absorption band between 200 and 340 nm is due to high valence or tetrahedral coordination of the transition metal ions in the alkali borate glass which agreed with the data reported in^64,65^. The nonlinear behavior of the position of the edge at around 340 nm was found to be compatible with the N4 values represented in Fig. 5. The UV absorption of glasses is considered to be influenced by both internal and external factors such as the electronic transitions, which are primarily caused by the addition of dopants and are influenced by the glass structure and chemical bonding^66^. Also, the change in the position of the absorption edge is due to the variation of oxygen bonding in the glass network^67^. The addition of metal cations such as Pb, Zn, Cd, and others affects the network formation of B_2_O_3_ and SiO_4_. These additives also act as a network modifier and a nucleating agent for glass crystallization. As a result, the optical properties of borate glasses have changed significantly^68^.

**Figure 7:**
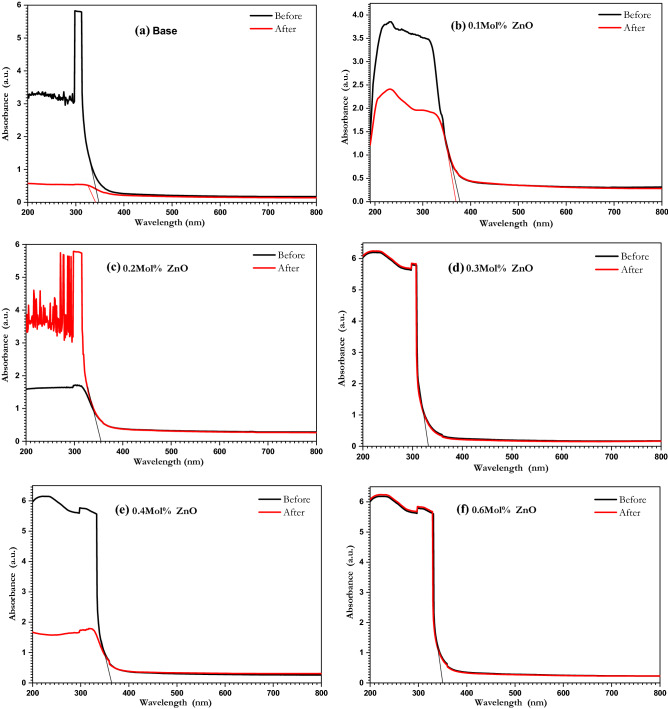
UV/vis absorption spectra of ZnO doped borate glasses before and after 30 min of laser irradiation.

Using a diode laser with a wavelength of 375 nm and 150 mW power the ZnO-doped borate glasses were irradiated for 30 min at room temperature. Figure 7a–f represents the absorption spectra for ZnO-doped borate glasses before and after the irradiation process. It can be figured that there was an absorption band in the UV region and no visible bands were observed for ZnO-doped borate glasses before and after the irradiation process as can be seen in Fig. 7a–f, only a change in the absorption intensity for the absorption band in the UV region and a small shift to the edge wavelength λ_edge_ were observed. The values of the λ_edge_ and direct optical energy gaps were calculated using Tauc plots and the Mott-Davis model ${\left(\alpha h\upsilon \right)}^{2}=B\left(h\upsilon -{E}_{g}\right)$^69,70^ are listed in Table 2.

**Table 2:** Optical energy gap before and after laser irradiation for ZnO doped borate glasses.

Sample	Before		After	
	${\uplambda }_{{{\text{edge}}}}$(nm)	${\text{E}}_{{\text{g}}}^{{{\text{opt}}}}$(eV)	${\uplambda }_{{{\text{edge}}}}$(nm)	${\text{E}}_{{\text{g}}}^{{{\text{opt}}}}$(eV)
Base	348.03	3.56	338.73	3.66
0.1 mol% ZnO	367.3	3.38	378.29	3.28
0.2 mol% ZnO	354.81	3.49	354.81	3.49
0.3 mol% ZnO	334.08	3.71	334.08	3.71
0.4 mol% ZnO	364.11	3.41	364.11	3.41
0.6 mol% ZnO	350.36	3.54	350.36	3.54

**Figure 8:**
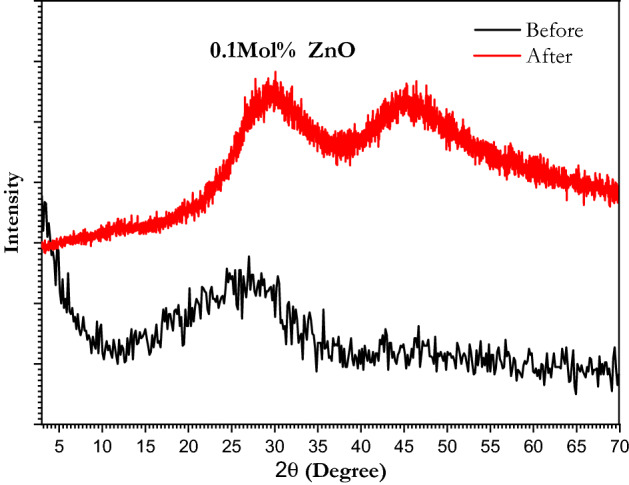
X-ray diffraction pattern of borate glass doped with o.1 mol% ZnO before and after 30 min of laser irradiation.

XRD was performed for o.1mol% ZnO doped sample and it’s obvious that clearly defined diffraction peaks were absent confirming the samples' glassy nature and ruling out the possibility of long-range atomic organization^48^. Figure 8 showed that before and after laser irradiation as there were two broad humps after laser irradiation around 29° and 46° were detected such humps distinguish non-crystalline solids (amorphous solids). Therefore, it can be stated that all studied samples are short-range order solids (glass solids).

### Cytotoxicity assay

It is necessary to evaluate the cytotoxicity of the material used in bio-applications. The culture of normal human skin fibroblasts (HSF) was used and investigated the influence of the glass with different concentrations on these cells.

Glass efficiency and potency in cells exposed to a drug (glass) are commonly evaluated using drug (glass) dose–response assays (e.g. MTT assay), and then the IC50 (half maximum inhibitory concentration) is estimated. With cell-based cytotoxicity studies, the 50 percent inhibitory concentration (IC50) is commonly employed to measure drug potency^71^. Zinc ions have also been linked to a variety of physiological activities, such as cell proliferation^72^. The result of cell viability was determined for the undoped and 0.6 mol% ZnO doped borate glasses Table 3. From the data in the table starting from 50 to 1.5625 μM of the glass material where a low concentration of each glass (1.5625 μM) gives a high percentage of viable cells, and also, they are non-toxic. After increasing the concentration of the glass material, the values of cell viability are still good compared to cell viability values in^73,74^.

**Table 3:** Values of cell viability of the undoped and 0.6 mol% ZnO doped borate glasses with different concentrations of each glass.

Glass	Cell viability %					
	50	25	12.5	6.25	3.125	1.5625
Base	89.28877	95.11568	96.11568	98.9709	101.1705	102.6281
	88.94773	94.14567	95.74464	97.34362	100.3436	103.1722
0.6 mol% ZnO	74.72151	82.26221	87.97601	90.48843	93.91602	95.45844
	83.69494	87.89289	91.52014	97.8329	99.11911	101.8029

**Figure 9:**
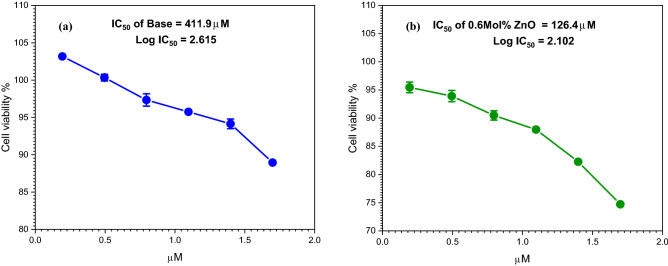
Effect of different amounts of (**a**) undoped glass, (**b**) 0.6 mol% ZnO doped borate glass on cell viability assay as determined from the absorbance at 570 nm and corresponding IC50 values.

**Figure 10:**
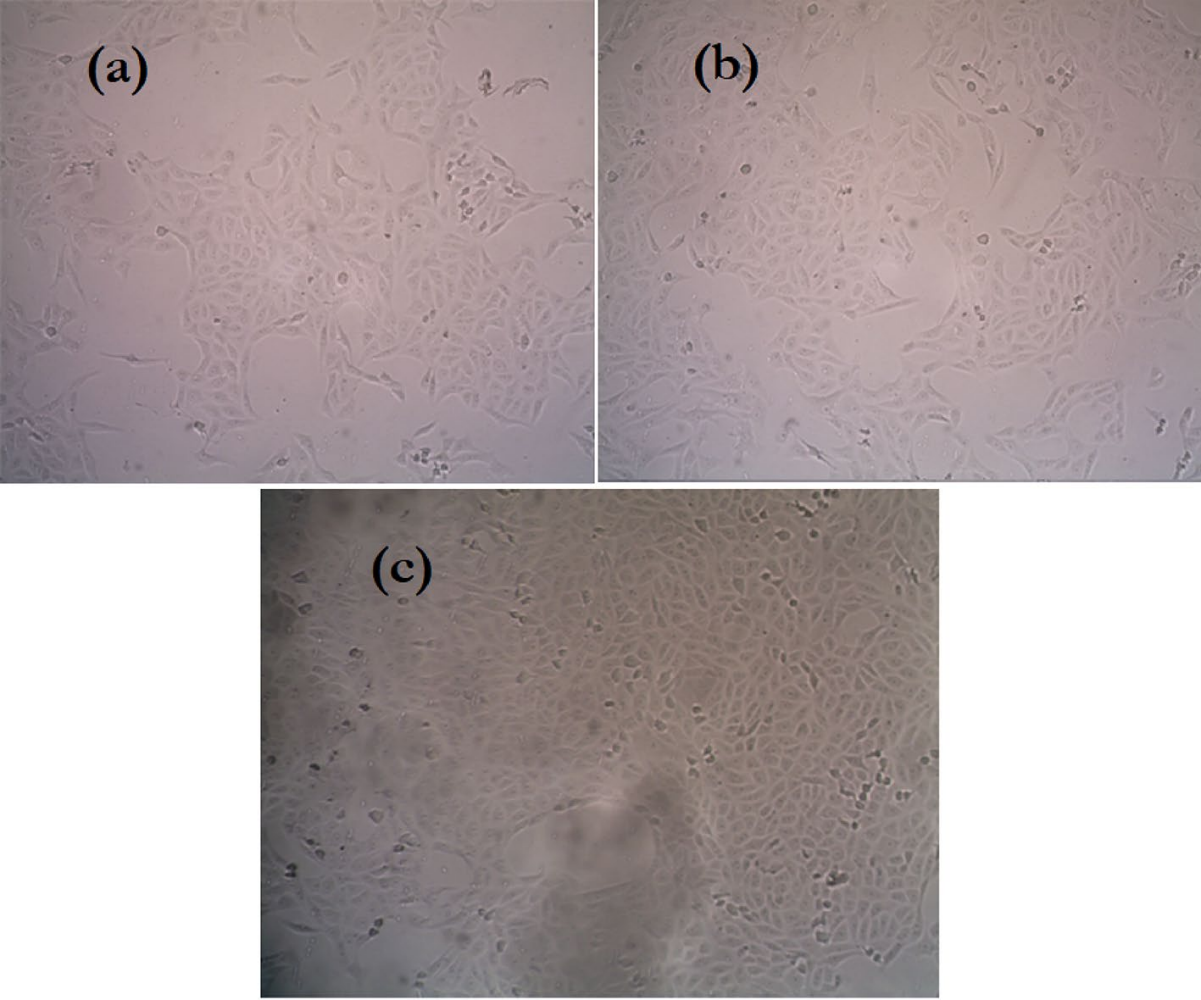
The optical microscope image of (**a**) undoped glass, (**b**) 0.6 mol% ZnO doped borate glass after cytotoxicity tests compared to (**c**) control sample.

Figure 9a,b represent the dose–response curves cytotoxicity assay of the undoped and 0.6 mol% ZnO doped borate glasses with different concentrations incubated in cell culture and showed the half-maximal inhibitory concentration (IC50). The standard error of the mean is represented by the error bars. Prasad S^75^, investigated in vitro cell proliferation using the MTT test on the base glass (BG0B) selected from the SiO_2_–Na_2_O–CaO–P_2_O_5_ system (S53P4 glass), as well as various modified glass compositions generated by replacing SiO_2_ in the base glass composition by B_2_O_3_ (BG1B, BG2B, and BG3B). It demonstrates that the cell proliferation was better on the B_2_O_3_-modified glasses (BG1B, BG2B, and BG3B) compared to the base glass (BG0B). It’s founded that cell growth was better on the B_2_O_3_-modified glasses (BG1B, BG2B, and BG3B) when compared to the base glass (BG0B). According to Balasubramanian et al.^76^ adding boron to bioactive glasses in various quantities has substantial effects on glass structure, glass processing characteristics, biodegradability, biocompatibility, bioactivity, and cytotoxicity. For bone and soft tissue engineering, various compositions of boron-doped, borosilicate, and borate glasses, are being studied. After cytotoxicity tests, the optical microscope image of the cells was taken (Fig. 10a–c). The morphology of cells that extend over the dish surface was not changed in the samples that showed low cytotoxicity as compared to the control sample. The morphology of the cells was approximately similar compared to the control sample, where the pulp cells indicated the survival cells and the rounded or shrunk cells indicated dead cells. It’s concluded that non-toxic behavior is exhibited by the prepared glasses compared to the control sample cells. Also, it is appropriate to be used for human tissue with no harmful effects.

## Conclusions

In this study, ZnO doped borate glasses with a composition of 6Na_2_O + 12K_2_O. + 5MgO + 20CaO + 4P_2_O_5_ + (53-x) B_2_O_3_ + xZnO, (0 ≤ x ≤ 0.6 mol%) were synthesized by traditional melt quenching technique. XRD study showed a high degree of amorphous structure for all samples. The formation of borate glass and the interaction with ZnO nanoparticles were indicated successfully by FTIR spectroscopy. Deconvolution analyses were applied to analyze the collected FTIR spectral data and showed a slight change in N4 coordinated boron but wasn’t noticeable due to the minor addition of zinc oxide. The incorporation of TMI was found to produce BO_3_ and BO_4_ structural units by shattering the boroxol (B_3_O_6_) ring, according to Fourier transform infrared (FTIR) spectra. UV–Vis optical properties were applied, and the optical energy gap was found to be around 3.4 eV. A highly intense band in the UV region in the range between 200 and 270 nm was noticed and found to be due to unavoidable trace elements introduced by raw materials. After the laser irradiation process, the optical energy gap was nearly similar for all samples but there was a change in the absorption intensity. The XRD pattern showed a change in the structure after the irradiation process, which indicate the short-range order of the investigated glass. Good cell viability was found for 0.6 mol% ZnO doped borate glass compared to the undoped glass after using the MTT assay method, the value of IC_50_ was decreased from 411.9 μM for borate glass to 126.4 μM for 0.6 mol% ZnO doped borate glass. As a result of that, and after future study on biodegradation and activity, ZnO-doped borate glasses with nominal composition is recommended for in vitro and in vivo bio applications.

## Data Availability

No data was used for the research described in the article. The data presented in this study are available in the article.
